# Whole Genome Sequencing-Based Comparison of Food Isolates of *Cronobacter sakazakii*

**DOI:** 10.3389/fmicb.2019.01464

**Published:** 2019-07-02

**Authors:** Mohamed A. Aly, Konrad J. Domig, Wolfgang Kneifel, Erik Reimhult

**Affiliations:** ^1^Department of Nanobiotechnology, Institute for Biologically Inspired Materials, University of Natural Resources and Life Sciences (BOKU), Vienna, Austria; ^2^Department of Food Science, Faculty of Agriculture, Ain Shams University, Cairo, Egypt; ^3^Department of Food Science and Technology, Institute of Food Science, University of Natural Resources and Life Sciences (BOKU), Vienna, Austria

**Keywords:** *Cronobacter sakazakii*, WGS, pathogenicity, biofilm formation, desiccation, multidrug-resistant

## Abstract

*Cronobacter sakazakii* is an emerging foodborne pathogen, which is linked to life-threatening infections causing septicemia, meningitis, and necrotizing enterocolitis. These infections have been epidemiologically connected to ingestion of contaminated reconstituted powder infant formula. Even at low water activity *C. sakazakii* can survive for a long time; it is capable of protective biofilm formation and occasionally shows high virulence and pathogenicity even following stressful environmental conditions. Hence it is a challenging task for the food industry to control contamination of food ingredients and products through the entire production chain, since an increasing number of severe food-related outbreaks of *C. sakazakii* infections has been observed. The seemingly great capability of *C. sakazakii* to survive even strict countermeasures combined with its prevalence in many food ingredients requires a greater in depth understanding of its virulence factors to master the food safety issues related to this organism. In this context, we present the whole genome sequence (WGS) of two different *C. sakazakii* isolated from skimmed milk powder (C7) and ready-to-eat salad mix (C8), respectively. These are compared to other, already sequenced, *C. sakazakii* genomes. Sequencing of the *fusA* allele revealed that both isolates were *C. sakazakii*. We investigated the molecular characteristics of both isolates relevant for genes associated with pathogenesis and virulence factors, resistance to stressful environmental conditions (e.g., osmotic and heat), survival in desiccation as well as conducted a comparative genomic analysis. By using multi-locus sequence typing (MLST), the genetic type of both isolates is assessed and the number of unique genes is determined. DNA of *C. sakazakii* C8 is shown to hold a novel and unique sequence type; the number of unique genes identified in the genomic sequence of *C. sakazakii* C7 and C8 were 109 and 188, respectively. Some of the determined unique genes such as the *rhs* and *VgrG* genes are linked to the Type VI Secretion System cluster, which is associated with pathogenicity and virulence factors. Moreover, seven genes encoding for multi-drug resistance were found in both isolates. The finding of a number of genes linked to producing capsules and biofilm are likely related to the observed resistance to desiccation.

## Introduction

*Cronobacter sakazakii* is regarded as an opportunistic foodborne pathogen, which can cause meningitis, bacteremia and necrotizing enterocolitis, particularly in infants (Drudy et al., 2006; Aly et al., 2018). Neonates have suffered from life-threatening infections of *C. sakazakii* within the first 28 days after birth (Mullane et al., 2007). Also, older infants have suffered fatality rates ranging from 40 to 80% and even immune-compromised adults have suffered fatalities (Nazarowec-White and Farber, 1997; Tsai et al., 2013). *Cronobacter* is one of the costliest foodborne pathogens due to the debilitation of survivors and loss of life. The cost of each case is estimated to be approximately 1 million USD (Minor et al., 2015). In epidemiological studies, infections in infants and neonates have been connected to ingestion of reconstituted powder infant formula contaminated with *C. sakazakii* (Hunter et al., 2008). *Cronobacter* survives under desiccated conditions in powdered infant formula even for extended durations of several months (Bennour Hennekinne et al., 2018). Generally, *C. sakazakii* can adapt to different adverse environments. It shows an unusual resistance to acid stress (ability to live in pH < 3.9), heat, desiccation (with surviving at a water activity of 0.118) and osmotic stress (continues to grow at 10% NaCl) (Fakruddin et al., 2014). *C. sakazakii* was shown to produce a capsular form or biofilm layer to protect itself from dry conditions. This helps to explain the high survival capability of *C. sakazakii* in infant powder milk (Hurrell et al., 2009). In addition, *C. sakazakii* can resist some antibiotics, thereby raising important issues regarding antibiotic therapies used to combat this organism (Kilonzo-Nthenge et al., 2012; Xu et al., 2015). For example, the resistance gene of colistin (*mcr-1*) was reported in *Cronobacter* (Liu et al., 2017). Further investigations of genes encoding for antibiotic resistance in *Cronobacter* isolates as well as genes responsible for its marvelous ability to survive the extreme environments in the food production chain are therefore of high priority for public health studies.

However, the mechanisms of pathogenicity in *C. sakazakii* remain largely unknown. It is known that after humans are infected by *C. sakazakii*, it can infect intestinal epithelial cells; from there, it invades the brain to infect human brain microvascular endothelial cells (HBMEC), confirming a route by which to cause meningitis (Giri et al., 2012). In this context, the outer membrane proteins A *(OmpA*) and X (*OmpX*) play important roles to explain how *Cronobacter* efficiently can adhere to and invade HBMEC, Caco-2, and INT-407 cells (Townsend et al., 2008; Kim et al., 2010; Holý et al., 2019). Strains expressing *OmpA* also resist killing and multiply in dendritic cells (Mittal et al., 2009; Holý et al., 2019). Moreover, *C. sakazakii* has been demonstrated not only to survive but also to replicate in macrophages (Townsend et al., 2007). Three putative virulence genes hemolysin (*hly*), plasminogen activator (*cpa*), and siderophore interacting protein (*sip*) were identified in *Cronobacter* spp. isolates (Cruz et al., 2011). Recently, multi-locus sequence typing (MLST) of 7 housekeeping genes was used to describe the diversity of the genus of *Cronobacter* from different sources (Joseph and Forsythe, 2012). Alleles known as a sequence type (ST) are determined using MLST schemes that interpret the nucleotide sequence data from a number of conserved housekeeping genes (Joseph and Forsythe, 2012); these enable comparisons and grouping of strains (Joseph and Forsythe, 2012). For example, *C. sakazakii* ST4 is predominantly connected with meningitis of neonates, while *C. sakazakii* ST1, 4, 8, and 12 strains are considered pathovars related to human illnesses (Joseph and Forsythe, 2011; Joseph et al., 2012b; Masood et al., 2015; Ogrodzki and Forsythe, 2017). *Cronobacter* spp. possess ubiquitous presence and have been isolated from cereals, rice, flour, dairy, herbs, medicinal plants, spices, meat, vegetables, and fruits (Cetinkaya et al., 2013; Ye et al., 2014). However, it is of interest to note that ST4 strains usually have been isolated from powdered infant formula and from milk powder processing factories (van Acker et al., 2001; Siqueira Santos et al., 2013; Sonbol et al., 2013; Gicova et al., 2014). Recently, efforts have been made to develop rapid detection schemes for *Cronobacter* species in food samples (Aly et al., 2018, 2019; Pan et al., 2018). Such technologies would greatly enhance food safety by allowing for continuous monitoring of contamination in the food chain.

Pulsed-field gel electrophoresis (PFGE) has long been considered the gold standard for molecular typing of the pathogens linked to outbreaks (Stranden et al., 2003; Alsonosi et al., 2015). However, PFGE has many limitations, including being incapable of distinguishing strains of highly clonal bacteria that are unrelated, as is the case for *Cronobacter* species (Alsonosi et al., 2015). Therefore, FoodNet is moving from PFGE to whole genome sequencing (WGS) for epidemiological investigations of foodborne outbreaks (Scharff et al., 2016; Nadon et al., 2017). WGS offers a more detailed resolution of how closely bacterial isolates are related than PFGE does; it additionally provides insights concerning the MLST, pathogenicity genes, as well as a complete molecular characterization of strains (Kozyreva et al., 2016; Forsythe, 2018).

Kozyreva et al. (2016) recently reported that WGS could be used to confirm the *Salmonella enterica* serovar Poona outbreak in California. Lytsy et al. (2017) examined the applicability, resolution, and reliability of PFGE, MLST, and WGS in three outbreaks in Sweden that occurred 2013–2015 for vancomycin-resistant enterococci. This study led to the recommendation to use WGS-ANI analysis for epidemiological identification of vancomycin-resistant enterococci (Lytsy et al., 2017). Publicly accessible genomic sequence data and the tools utilized to examine these data are now ubiquitous in biological studies, with the food safety research area as no exception (Taboada et al., 2017). Currently available bioinformatics tools for comparative genomic analysis, such as pan-genome analysis, have been employed to characterize the entire gene repertoire of bacterial species (Medini et al., 2005). In molecular biology, the pan-genome is defined as the complete set of non-orthologous genes present in species, consisting of the core and accessory genomes, i.e., sets of genes that are present in all strains and unique to single strains, respectively (Kim et al., 2015). Moreover, this analysis confirms how many new genes can be determined from newly sequenced genomes.

In this work, we use WGS to compare the sequences of *C. sakazakii* isolated in Austria with other previously published genomes to obtain additional insights into their genetic makeup. We focus our study on parts of the genome that support our understanding of previous observations of pathogenicity and long-term persistence of *C. sakazakii*. In detail, we investigate and characterize some of the unique genes detected and associated with virulence and pathogenicity mechanisms, resistance to stressful conditions, biofilm formation, and multidrug-resistance.

## Materials and Methods

### Bacterial Isolates and Genome Sequencing

The two *Cronobacter* isolates used in this work named *C. sakazakii* C7 and C8 were isolated from skimmed milk powder and ready-to-eat food (salad mix) samples, respectively. The isolates were obtained in Austria and confirmed as described previously (Aly et al., 2019). They were identified by the *rpo*B gene (Stoop et al., 2009). Furthermore, *fusA* sequences were queried in MLST databases^1^ to confirm their belonging to the *Cronobacter* species. The bacteria were cultured as described in our earlier work (see text footnote 1; Aly et al., 2019). The genomic DNA (gDNA) of both isolates was extracted and purified using the bacteria genome kit (peqGOLD Bacterial DNA Kit, VWR International GmbH, Germany), according to the guidelines from the manufacturer (Aly et al., 2019). Optical density measurements of the gDNA was performed in a Qubit Fluorometric Spectrophotometer (Life Technologies, Wilmington, DE, United States). Libraries were generated by enzyme fragmentation and constructed using the NEBNext Fast DNA-Library for Ion Torrent sample kit preparation (New England Biolabs, Ipswich, MA, United States) following the manufacturer’s instructions. The fragmented gDNA was end-repaired and ligated to the specific adapters and individual barcodes. The quality control of the gDNA libraries was determined with the Agilent Bioanalyzer 2100. The enriched libraries were amplified using emulsion polymerase chain reaction (ePCR, Ion PI^TM^ Hi-Q^TM^ OT2 200 kit), following the manufacturer’s instructions (Thermo Fisher Scientific, Inc.). Ion PI^TM^ Chip kit v3 chips were employed on the Ion Torrent Proton (Thermo Fisher Scientific, Inc.) platform for the WGS.

### Bioinformatics

The single end sequencing reads generated from the Ion Torrent sequencing were trimmed using the Trimmomatic Tool version 0.36 (Bolger et al., 2014) and filtered by the quality control step of the coupling pipeline in a FASTAQ file format. *De novo* assembly of high-quality reads is performed with SPAdes (version 3.9.0) assembler (Bankevich et al., 2012). The Quast software was used to evaluate the *de novo* assembly results. The results were generated on a scaffold based on the number of contigs, GC-depth analysis, coverage analysis of assembly, and comparison and N50 to ensure its quality (Gurevich et al., 2013). Scaffolds greater than 500 bp in sequence length were used for downstream analysis. Next, genome annotation (DNA annotation) was conducted through the “Rapid Annotated using Subsystem Technology” (RAST) with server pipeline (Aziz et al., 2008; Overbeek et al., 2014) in a FASTA file format. RNA gene and protein-coding sequencing (CDS) was used to assign functions and determine the presence of subsystems in the genome. Comparative genome to genome analysis was evaluated in the SEED viewer as previously reported (Overbeek et al., 2014). Homologs of conserved genes in both isolates were identified using the annotation of the NCBI GeneBank of *C. sakazakii* ATCC BAA-894 (GCA_000017665.1) as the reference genome. The analysis of bacterial pan-genome was performed by the ultra-fast bacterial pan-genome analysis pipeline (BPGA) tool (Chaudhari et al., 2016), and GView Server (Petkau et al., 2010) using the retrieved published complete genomes from the NCBI database including those of, *C. sakazakii* ES15 (GCF_000263215.1), *C. sakazakii* BAA-894 (GCA_000017665.1), *C. sakazakii* NCTC-8155 (GCF_001277275.1), and *C. sakazakii* Sp291 (GCF_000339015.1). These four genomes were compared to the genomic sequence in our study *C. sakazakii* C7 and C8. BLASTP search with functional annotation of the unique and core genes was performed by the BLAST2GO analysis pipeline (Götz et al., 2008), applying the default settings for the BLAST search expectation value (E value) of 1.0 × 10^–3^. Phage-associated gene region clusters in the assembly sequence of *C. sakazakii* C7 and C8 isolates were identified using the PHASTER server (Arndt et al., 2016). Three scenarios for the wholeness of the identified phage-associated region clusters were assigned according to how many proteins/genes of a known phage the region involved (Dennis et al., 2011): intact (>90%), questionable (70–90%) and incomplete (<70%). Antibiotic resistance genes in the genome assembled for *C. sakazakii* were detected by a search against a local antibiotic resistance gene sequence from the CARD (Comprehensive Antibiotic Resistance Database) database (Jia et al., 2017).

### O-Serotype Determination Analysis

The *gnd* and *galF* loci gene clusters are specific for the O-serotype region; they were identified from the BLAST gDNA sequences by the BIGSdb pipeline tools in the PubMLST typing database (see text footnote 1; Jolley and Maiden, 2010; Ogrodzki and Forsythe, 2015).

### Multi-Locus Sequence Typing (MLST)

Briefly, MLST was utilized to query each genome for all known alleles at each locus by homology check, with new alleles recognized and afforded with a unique allele number. MLST of *Cronobacter* was executed by submitting genome sequences to the PubMLST typing database for *Cronobacter* (see text footnote 1; Larsen et al., 2012). The 7-loci MLST profiling of *Cronobacter* being glutaminyl tRNA synthetase (*glnS*), glutamate synthase large subunit (*gltB*), ATP synthase b chain (*atpD*), DNA gyrase subunit B (*gyrB*), phosphoenolpyruvate synthase A (*ppsA*), translation initiation factor IF-2 (*infB*), and elongation factor G (*fusA*). Speciation of *Cronobacter* spp. was obtained by a phylogenetic analysis sequence of the *fusA* allele (Joseph et al., 2012a; Forsythe et al., 2014; Forsythe, 2015).

The whole genomes were submitted to NCBI under the *C. sakazakii* complete genome bioproject number PRJNA510032 and biosample accession numbers are SAMN10593105 and SAMN10593106 for *C. sakazakii* C7 and C8, respectively.

## Results and Discussion

### General Genome Properties

**FIGURE 1:**
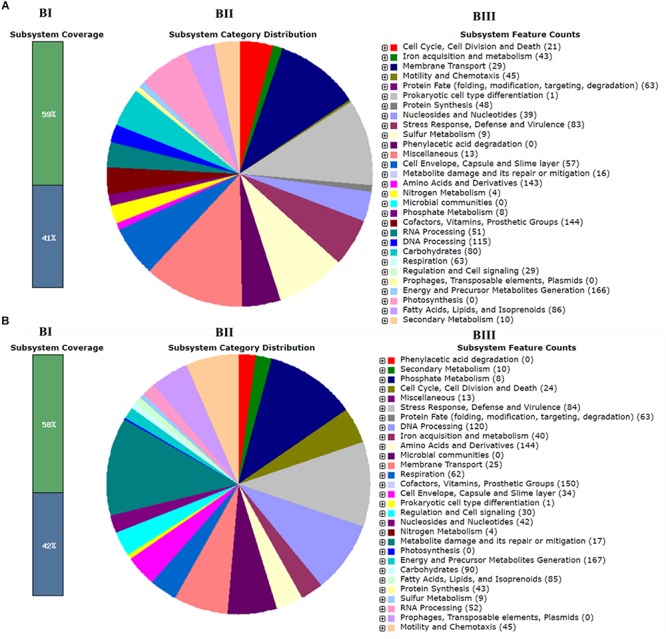
Subsystem category distribution of *Cronobacter sakazakii* based on the SEED database. **(A)** C7 and **(B)** C8. Bar chart (BI) shows the percentage of subsystem coverage (green bar corresponds to the percentage of proteins involved). The pie chart (BII) with legend (BIII) shows the fraction and count (parenthesis in legend) of each subsystem feature.

**FIGURE 2:**
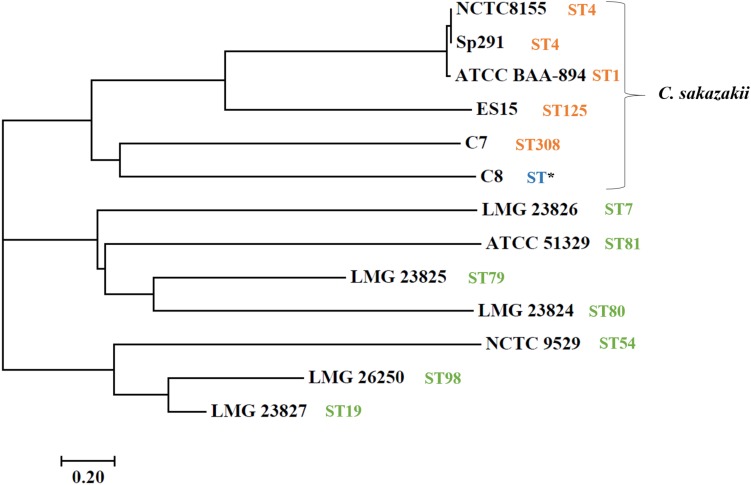
Neighbor-joining tree of *C. sakazakii* isolated from food, compared with seven representative *Cronobacter* genus type strains. Accession numbers (NCBI GenBank) of type strains: *C. sakazakii* ES15 (GCF_000263215.1), *C. sakazakii* BAA-894 (GCA_000017665.1), *C. sakazakii* NCTC-8155 (GCF_001277275.1), *C. sakazakii* Sp291 (GCF_000339015.1), *C. universalis* NCTC 9529 (NZ_CP012257), *C. condimenti* LMG 26250 (NZ_CP012264), *C. malonaticus* LMG 23826T (NZ_CP013940), *C. turicensis* LMG 23827 (NC_013282), *C. dublinensis* subsp. *lausannensis* LMG 23824 (NZ_AJKY00000000), *C. dublinensis* subsp. *lactaridi* LMG 23825 (NZ_AJKX00000000), and *C. muytjensii* ATCC 51329 (NZ_CP012268). The tree was created in MEGA version 7. Sequence type (ST) information was obtained from MLST website (http://pubmlst.org/cronobacter/). Asterisks (^*^) indicate novel sequence.

Whole-genome sequencing showed a total of 2,193,827,360 bp, and 2,265,893,859 bp with single-end reads from gDNA of C7 (milk powder *C. sakazakii* isolate) and C8 (ready-to-eat salad *C. sakazakii* isolate). After quality control, 1409 Mbp of high quality reads were obtained for C7 and 1472 Mbp were obtained for C8. These were kept for *de novo* assembly, which yielded a total of 64 scaffolds with total scaffold N50 of 344,291 bp for C7 and 61 scaffolds with total scaffold N50 of 436,880 bp for C8. The genomic features of C7 and C8 are summarized in Table 1 and functionally annotated by the RAST server (Figure 1 and Supplementary Table S1). Figure 2 illustrates this phylogenetic analysis of WGS using the MEGA 7 tools. The distances between branches in Figure 2 were calculated using the Maximum Composite Likelihood method (Tamura et al., 2004; Kumar et al., 2016). The phylogeny analysis of the seven species members in genus of *Cronobacter* strains revealed that the ST of *C. sakazakii* strains formed their own distinct cluster.

**TABLE 1:** General genome features of both *Cronobacter sakazakii* isolates.

**Feature**	**C7**	**C8**
Genome size (bp)	4,388,331	4,488,633
GC content (%)	57.1	56.9
Number of CDS	4074	4192
Total number of RNA	83	81

**FIGURE 3:**
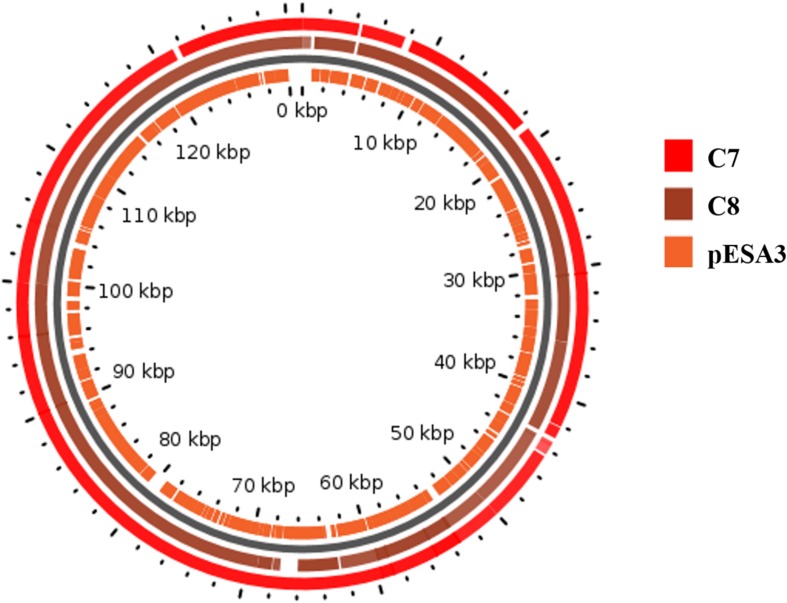
Map of the plasmids from *C. sakazakii* C7 and C8. The rings show BlastN comparison with homologous plasmids from *C. sakazakii* ATCC BAA-894 plasmid pESA3. The map was drawn in GView (https://server.gview.ca/).

A map showing the BlastN comparison of the plasmids from *C. sakazakii* C7 and C8 with homologous plasmids from *C. sakazakii* ATCC BAA-894 plasmid pESA3 is shown in Figure 3. Comparative analysis by RAST pipelines of the WGS assemblies with that of the putative virulence plasmid showed the existence of plasmid genes P1 (126 kbp), pESA3 (131 kbp), and pCTU1 (138 kbp) (see Supplementary Table S2). *In silico* analysis of plasmid P1, pESA3, and pCTU1 showed that these plasmids possess chromosome (plasmid) partitioning proteins *parAB* genes immediately upstream of plasmid replication protein *repA*. The *C. sakazakii* C7 and C8 strains share some of the alleles present in pESA3 and pCTU1 as described by Franco et al. (2011). This plasmid contains many virulence factors, including iron acquisition systems (*iucABCD*/*iutA*), protease VII (*Omptin*), ABC transporter (iron.B12.siderophore.hemin), aerobactin siderophore and the T6SS gene cluster. The *C. sakazakii* C7 and C8 strains harbor a plasmidborne T6SS gene cluster such as *VgrG* (valine-glycine repeat G protein) as described by Franco et al. (2011). These results suggest that the virulence factors cluster in the sequenced genome, as also observed in previous studies (Kucerova et al., 2010; Franco et al., 2011; Yan et al., 2013; Jang et al., 2018; Kadlicekova et al., 2018). A site-specific integrase was detected in the *in silico* analysis of each plasmid (C7 and pESA3 at position 66 Kbp) and was found to belong to integrases in an operon arrangement with four genes encoding a hypothetical protein. These genes were absent in the C8 plasmid. The same genes were previously observed on the plasmid pESA3 by Franco et al. (2011). A channel-forming transporter/cytolysin activator of the *TpsB* family gene was found at 80 Kbp. It was clustered in the plasmid sequences of C7 and C8 but was absent in plasmid pESA3. Also, at 130 Kbp a gene encoding a hypothetical protein was found within both the C7 and C8 genomes. In previous studies, *C. sakazakii* strains have been found to harbor plasmids such as pSP291–3 (Yan et al., 2013), pESA2 (Kucerova et al., 2010), pCTU2, and pCTU3 (Stephan et al., 2011; Yan et al., 2013). However, we did not find these plasmids in the strains that we analyzed.

### Predicted O-Serotype of *Cronobacter* Isolates

The prediction of O-serotype was based on the presence of flanking genes *gnd* and *galF* on the DNA sequence following the approach of Ogrodzki and Forsythe (2015). In this study, the *C. sakazakii* C7 isolated from skimmed milk powder was representative of O-antigen serotype (*Csak_*O2) gene clusters located between the *gnd* and *galF*. However, only 17 serotypes, all O-serotypes, have been determined in *Cronobacter* spp. (Mullane et al., 2008; Jarvis et al., 2011; Sun et al., 2012). The *C. sakazakii* C8 isolate from ready-to-eat food represents an unknown serotype; there have been no O-antigen genes identified for these flanking loci. This demonstrates that the current standards for molecular serotyping are not adequate and additional protocols for serotyping should be developed to enable serotyping of all *C. sakzakii*. Additional data on the distribution of serotypes are also required to define whether molecular serotyping is a helpful alternative for epidemiological surveillance of the *Cronobacter* genus.

### Multilocus Sequence Typing (MLST)

Multilocus sequence typing is a method to distinguish strains in the *Cronobacter* genus, using the seven-loci MLST (Forsythe et al., 2014). It has been reported that the *fusA* allele sequence is matched with the phylogeny sequence and could be utilized for speciation of the *Cronobacter* genus (Joseph et al., 2012a). The resting six alleles could then be defined for seven-loci MLST profiling. In the current study, the MLST results for *C. sakazakii* C7 and C8 isolates are summarized in Table 2. The MLST revealed C7 to be of ST308. Surprisingly, C8 isolated from ready-to-eat food was found to be a new unique ST with novel alleles [*gltB*, *infB*, and *ppsA*]. These are single nucleotide substitution variants when compared with their most homologous alleles. This finding combined with the few investigations to date of the genetic populations of *C. sakazakii*, indicate that it is essential to recognize more STs to aid in epidemiological investigations and risk assessments of this pathogen.

**TABLE 2:** Clustering of *Cronobacter* sequence type by multilocus sequence typing.

**Isolates**	**Locus**	**Identity**	**Coverage**	**Alignment length**	**Allele length**	**Gaps**	**Allele**	**Sequence type (ST)**
C7	atpD	100	100	390	390	0	atpD_16	308
	fusA	100	100	438	438	0	fusA_18	
	glnS	100	100	363	363	0	glnS_120	
	gltB	100	100	507	507	0	gltB_119	
	gyrB	100	100	402	402	0	gyrB_88	
	infB	100	100	441	441	0	infB_73	
	pps	100	100	495	495	0	pps_18	

C8	atpD	100	100	390	390	0	atpD_3	Unknown nearest STs: 348, 318, 654, and 641
	fusA	100	100	438	438	0	fusA_10	
	glnS	100	100	363	363	0	glnS_120	
	gltB	99.8028	100	507	507	0	gltB_59^*^	
	gyrB	100	100	402	402	0	gyrB_125	
	infB	99.5465	100	441	441	0	infB_236^*^	
	pps	99.1919	100	495	495	0	pps_321^*^	

^*^ alleles with less than 100% identity found (Novel allele, ST may indicate nearest ST).

### Comparative Genomic Analysis

#### Pan-Genome Analysis

**FIGURE 4:**
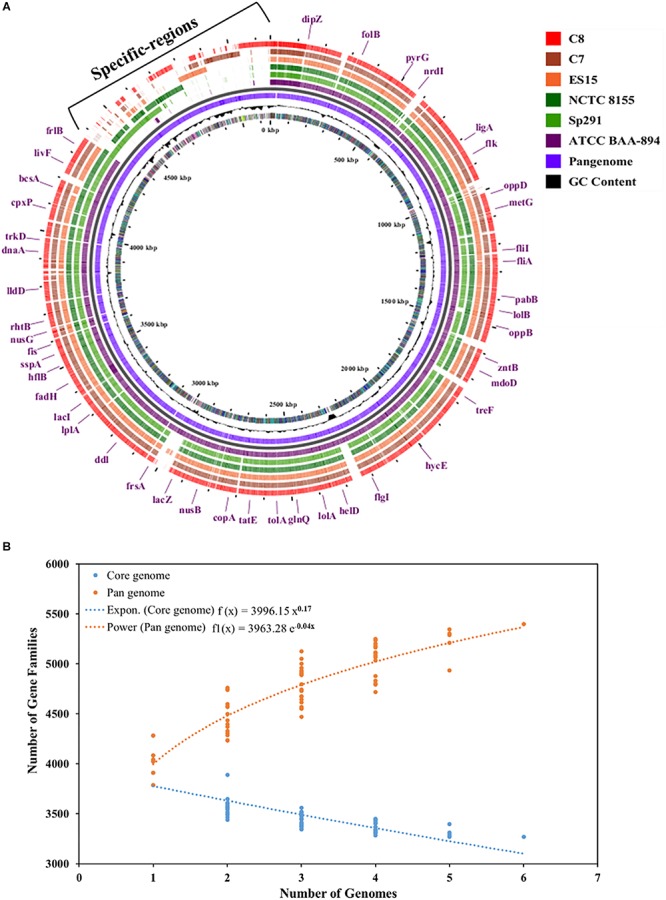
**(A)** Pan-genome of six *C. sakazakii* strains (4 database genomes and the 2 isolates) was visualized using the Gview server (Zheng and Huang, 2004; Petkau et al., 2010). The cut-off value was 80% of BlastN homology. The annotation indicates clusters of conserved genes with *C. sakazakii* (ATCC BAA-894). **(B)** Plot showing the pan- and core-genome of *C. sakazakii*. The overall number of genes or pan-genome (Orange) and shared or core-genome (Blue) of 6 *C. sakazakii* are shown in the plot. The pan-genome consists of 5399 genes, while the core-genomes are made up of 3268 genes.

The complete genomes of four different *C. sakazakii* strains and their annotation data have been published to date in the GenBank database. As illustrated in Figure 4A, the pan-genome analysis of these 4 complete genomes and those of the 2 new isolates, C7 and C8, presented here were conducted and strain specific-regions were obtained and visualized. An analysis by the BPGA tool was applied to the 6 *C. sakazakii* strains. First, the core- and pan-genomes and their sizes and trajectories were analyzed using the approach suggested by Knight et al. (2016). Second, the median counts were extrapolated employing two models: exponential regression described in Tettelin et al. (2005) and power-law regression described in Tettelin et al. (2008). The resulting extrapolations from the gene counts were normalized by the mean of the genome sizes of the respective sets to aid in the comparison and visualization of the fits (Figure 4B).

The pan-genome was made up of 5399 genes, and the *C. sakazakii* strains shared 3268 genes as core-genomes (Figure 4B and Supplementary Table S3A). The BGPA power law regression analysis identified the pan-genome of *C. sakazakii* strains as open (Supplementary Table S3B). The pan-genome is recognized to be “open” as long as new genes significantly increase the total repertoire for each new extra genome and “closed” when newly added genomes do not significantly add to the total repertoire of the genes (Guimarães et al., 2015). Mathematical modeling of the data implies that the pan-genome gene reservoir of unique genes will increase even after hundreds of newly sequenced genomes are added and therefore is considered to be open (Tettelin et al., 2005; He, 2015). The number of unique genes detected in *C. sakazakii* BAA-894 and *C. sakazakii* NCTC-8155 were 465 and 53, respectively (Table 3). The *C. sakazakii* C7 and *C. sakazakii* C8 have a total of 109 and 188 unique genes conserved in gDNA, respectively. The gene ontology (GO) of the unique genes in gDNA of *C. sakazakii* C7 revealed that they are rich in proteins associated with molecule binding, heterocyclic, and organic cyclic compound binding, hydrolase activity, drug and ion binding (Supplementary Tables S3C,D).

**TABLE 3:** Number of core, unique, accessory, and exclusively absent (from a particular strain) genes taken from the pan-genome analysis of 6 *C. sakazakii* strains.

**Genome no.**	**Organism name**	**Originally isolated from**	**No. of core genes**	**No. of accessory genes**	**No. of unique genes**	**No. of exclusively absent genes from a particular strain**
1	*C. sakazakii* ATCC -BAA-894	Powdered milk formula	3268	550	465	42
2	*C. sakazakii* C7	Skimmend milk powder	3268	535	109	13
3	*C. sakazakii* C8	Ready-to-eat food (salad mix)	3268	567	188	44
4	*C. sakazakii* ES15	Ground whole grain	3268	423	95	128
5	*C. sakazakii* NCTC-8155	Tin of dried milk	3268	764	53	1
6	*C. sakazakii* Sp291	Powdered infant formula	3268	678	95	24

#### Genotype of Antimicrobial Resistance (AMR)

The presence of multidrug-resistant genes in gDNA that may contribute to antimicrobial resistance was identified by BLAST searching the assembled sequence genome of *C. sakazakii* isolates against a local copy of CARD sequence data (Supplementary Table S4). As shown in Supplementary Table S4, seven antibiotic resistance genes were found: *msbA*, *emrR*, *H-NS*, *emrB*, *marA*, CRP, and *PBP3*. These provide resistance to multiple antibiotics, such as, beta-lactam antibiotics, tetracycline, a macrolide antibiotic, a fluoroquinolone antibiotic, penams, cephalosporin, and cephamycin. The genomes of both *C. sakazakii* isolates contained all these genes. Active efflux pumps provide a known mechanism for increased virulence by improving the survival of *Enterobacteriaceae* in the host’s gastrointestinal tract (Touze et al., 2004). Further, the efflux system enables the invasion of brain microvascular endothelial cells (Franke et al., 2003; Kucerova et al., 2010). Tetracycline-resistance was reported for *C. sakazakii* isolated from a freshwater Chilean salmon aquaculture farm (Miranda et al., 2003). Previous studies have shown that abuse of antibiotics in such environments and the presence of various antibiotic resistance (*mar*) operons may enable *Cronobacter* spp. to develop resistance to numerous different antibiotics (Kim et al., 2008; El-Sharoud et al., 2009; Chon et al., 2012). Hence, the large number of multidrug-resistant genes found here, with an unknown entryway to the *C. sakazakii* genome, suggests that further studies are required to define the extent of acquired antimicrobial drug resistance and an attempt to map the way it was acquired. Only by such efforts can we prevent the emergence of additional antimicrobial resistance or create early warnings of the emergence of such resistance.

#### Genes Involved in Resistance to Environmental Stress

The high survival rate of *C. sakazakii*, under extreme dessication (e.g., in milk powder, starch, or flour), low pH, heat and osmotically challenging environments, has so far not been examined at the molecular level. We performed WGS also to understand this unusual and challenging property of *C. sakazakii*. Supplementary Table S1 includes a number of genes associated with resistance to stressful environmental conditions identified in the genomic sequences of the *C. sakazakii* C7 and C8 isolates. Osmoregulation genes such as osmotically inducible protein (*OsmY*), transcriptional regulatory protein (*YciT*), aquaporin Z, hyperosmotic potassium uptake protein (*TrkH*) potassium uptake protein (*TrkA* and *TrkG*), *ProP*, Betaine aldehyde dehydrogenase (*otsA)*, Trehalose-6-phosphate hydrolase (*ostB*), and Glutathione-regulated potassium-efflux system protein (*KefB*, *KefC*, and *KefG*) were identified in both genomes of *C. sakazakii* isolates. In addition, heat/cold shock stress genes were also identified, such as *DnaJ* and *DnaK* suppressor proteins and the heat shock proteins (*YciM*) and (*GrpE)*. It is supposed that when *C. sakazakii* is exposed to low water activity conditions, it rapidly accumulates electrolytes to increase the internal osmotic pressure; this is the primary fast action by which it protects itself by counteracting the high external osmotic pressure in desiccated environments (Feeney and Sleator, 2011). Jang et al. (2018) also found genes encoding for proteins associated with osmotic response, such as Aquaprotein Z, *DnaJ*, *TreF* and *ProQ*. For example, *DnaJ* operates actively in the response to hyperosmotic and heat shock by preventing the aggregation of stress-denatured proteins. The physiological mechanisms of survival in desiccated environments are thought to include countermeasures to both primary and secondary desiccation stresses. Glycine betaine, trehalose and other osmoprotectants are used in adaptation strategies by xerotolerant organisms (Santos and Da Costa, 2002; Srikumar et al., 2019). For example, the osmoprotectants found in osmotically stressed *E. coli* are trehalose, glycine betaine proline, and carnitine (Dinnbier et al., 1988; Lehner et al., 2018; Srikumar et al., 2019). These previous reports describe two genes, *otsA* and *otsB*, that are part of the biosynthetic pathway for trehalose, which were transcriptionally highly up-regulated in *C. sakazakii* cells grown under xerotolerant conditions (Srikumar et al., 2019). The Glutathione-gated potassium-efflux systems (*Kef*) can also play an important role in desiccation tolerance by preventing extended exposure of bacterial cells to excess potassium ions (Du et al., 2018). The *proP* gene found in our isolates have been shown to be highly upregulated when the organism has been subjected to a desiccating environment. Deletion of the *proP* gene in *Salmonella* Typhimurium led to loss of viability during desiccation for long periods of time (Finn et al., 2013). We also identified the outer membrane protein W (*OmpW*) gene in the genomes of both *C. sakazakii* isolates. It was reported that the *OmpW* gene contributes to survival of *C. sakazakii* under osmotic and oxidative stress, as well as being associated with increased biofilm formation (Ye et al., 2018; Zhang et al., 2019). Furthermore, epoxide hydrolase was identified as a unique gene in the genome of *C. sakazakii* C7 (Supplementary Table S3C); it plays a vital role in the degradation of organic compounds and is potentially useful in enantioselective biocatalysis. Being able to survive in dried material is a known characteristic of *C. sakazakii* and contributing to its virulence. Previous investigations have also described that *C. sakazakii* is able to survive extreme temperatures, including heat-shock at 47°C and cold-shock at −20°C (Shaker et al., 2008; Chang et al., 2010; Gajdosova et al., 2011). The identified genes provide molecular links to these remarkable properties.

#### Genes Associated With Biofilm Formation

It has been demonstrated that *C. sakazakii* can produce biofilms on stainless steel, glass, polyvinyl chloride, polystyrene, silicone, and latex surfaces (Kim et al., 2006; Hurrell et al., 2009; Aly et al., 2019); these biofilms could not always be eliminated even by thorough washing procedures using disinfectants and water. This capability of biofilm formation by *C. sakazakii* makes it a more capable pathogen (Shi et al., 2017). We found genes linked to the ability to form biofilms in the genomes of both *C. sakazakii* isolates (Supplementary Table S1). These include genes for biosynthesis, such as colanic acid biosynthesis process, flagellar assembly protein (*FliH)*, flagellar protein (*FlgA-K*) and *FliCJLPQSTZ, FlhA-E* genes linked to the biosynthesis of flagella. Bacterial flagella have been shown to have multiple important roles in biofilm development (Wood, 2013; Kang et al., 2015). They provide cell motility, which is important during formation of biofilms as well as for dispersal bacteria from biofilms, and they play an important role in sensing and colonization of surfaces (Cammarota et al., 2012; Friedlander et al., 2015; Ratthawongjirakul et al., 2016). The *flgJ* gene is associated with the biosynthesis of a flagellum and it has been described that the presence of the *flgJ* gene led to increased ability to form biofilms (Ye et al., 2015; Bao et al., 2017a). A unique sequence annotated as filamentous hemagglutinin protein (FHA) was identified in the genome of isolate C7 (Supplementary Tables S2, S3C). In *Cronobacter* spp. this gene is present on the pCTU1 plasmid and it encodes a filamentous hemagglutinin (Franco et al., 2011). This gene is suggested to play a role in promoting bacterial aggregation and adhesion. In *Bordetella*, the filamentous hemagglutinin leads to adhesion both in a secreted and surface-associated form (Lambert-Buisine et al., 1998; Jacob-Dubuisson et al., 2000; Franco et al., 2011). In *Xanthomonas axonopodis*, genes encoding for filamentous hemagglutinin protein is needed for surface attachment, tissue colonization and linked to biofilm formation (Gottig et al., 2009). The *grxA* family glutaredoxin was identified as a unique gene in the genome of *C. sakazakii* C8 (Supplementary Table S3D); it is also implicated to play a vital role in biofilm formation. It was recently demonstrated in *E. coli* that *grxA* is associated with biofilm formation and with prolonging attachment periods following cell attachment to the substrate (Hancock and Klemm, 2007; Bhomkar et al., 2010). *E. coli* strains that were functionally deleted for *grxA* formed biofilms that had lower survival rates after exposure to metal ions (Harrison et al., 2007). Recently, the link between cellulose (a component of the biofilm matrix) production and biofilm formation ability of *C. sakazakii* was experimentally demonstrated (Hu et al., 2015; Aly et al., 2019).

*Cronobacter sakazakii* have been shown to have the gene cluster of *nanAKTR* to utilize sialic acid as a carbon source for bacterial growth (Joseph et al., 2013). We found that *C. sakazakii* C7 and C8 have the gene clusters *nanT, nanR*, and for the first time found *nanC*, for sialic acid metabolism. This unique capability to utilize sialic acid for biosynthesis during biofilm formation could be due to an adaptation to the milk powder environment, because milk contains sialic acid (Wang et al., 2001).

The genes associated with desiccation resistance were identified in both genomes; they include Capsular polysaccharide ABC transporter, Colanic acid capsular biosynthesis activation proteins *rcsA* and *rcsB*, Putative capsular polysaccharide transport protein (*YegH*) and Capsular polysaccharide genes cluster (*KpsCDES*). The ability to produce a heteropolysaccharide capsule by both *C. sakazakii* isolates can enhance their ability to form biofilms, which also contributes to their desiccation resistance. Recently, (Ogrodzki and Forsythe, 2015) reported that capsule-associated genes in a *C. sakazakii* clinical strain is a potentially important virulence trait linked to severe neonatal infection. Furthermore, colanic acid is known as a component of exopolysaccharides in the genus of *Cronobacter*, which promotes adhesion to different abiotic surfaces with increased resistance for environmental stress factors such as heat, acid, antibiotics and desiccation (Jung et al., 2013). Our observations here agree well with the biofilm formation ability under different conditions of both *C. sakazakii* C7 and C8 described in our earlier study (Aly et al., 2019). We emphasize that the two unique sequences annotated with filamentous hemagglutinin protein (C7) and *grxA* gene (C8) associated with the biofilm-forming ability have previously not been observed in *C. sakazakii*.

#### Pathogenicity and Virulence Factors

To define the conserved genes associated with pathogenicity, we identified the virulence factors of *C. sakazakii* C7 and C8 isolates (Supplementary Tables S1, S3). The genes of C7 and C8 associated with virulence can be classified into 5 categories. These are: adherence, invasion, secretion system, iron uptake, and toxins.

Starting with the adherence category, we found outer membrane protein A (*OmpA), FliR, fliC*, and *FlhA* adhesion genes in the genomes of both the C7 and C8 isolates. It has been reported that the *OmpA* gene led to improving cell adhesion of *C. sakazakii* and invasion of HBMEC (Nair et al., 2009), suggesting both isolates may invade HBMEC. The *FliR* gene is mainly responsible for the structural components of the flagellum. Flagella are implicated in many mechanisms of adhesion to host cells and pathogenicity; the *FliR* gene is therefore instrumental in both adherence and invasion (Du et al., 2016). The *fliC* gene has also been strongly associated with adherence and virulence traits of the pathogens (Dingle et al., 2011; Aldubyan et al., 2017; Holý et al., 2019). The *FlhA* gene is implicated as required for export of flagellin and therefore assembly of flagella; it therefore has been described to play a role in invasion into and adhesion to epithelial cells by various bacteria, such as *Pseudomonas aeruginosa, Bacillus thuringiensis and B. cereus* and *C. sakazakii* (Fleiszig et al., 2001; Ramarao and Lereclus, 2006; Du et al., 2016).

Additionally, identified genes that potentially can aid invasion and contribute to pathogenicity and virulence were annotated as efflux system component, zinc metalloprotease *YfgC* precursor, putative S-ribosyl homocysteine lyase encoded by the *luxS* gene and protein hemolysin III homologs. The silver and copper cation efflux system has also been implicated in facilitated invasion of HBMEC (Kucerova et al., 2010). Furthermore, the superoxide dismutase gene was present in the genome; it has been implicated in improved survival of bacteria in macrophages (Townsend et al., 2007; Bao et al., 2017b).

In the secretion system category, based on sequence analysis, we identified several putative genes of Type VI Secretion System cluster in the genomic sequence of both isolates. In addition, we identified unique sequences linked to the Type VI Secretion, such as the *rhs* gene in the genome of C7 and the *VgrG* gene cluster in the genome of C8. The Type VI Secretion System (T6SS) is a versatile protein secretion machinery able to immediately deliver protein toxins into eukaryotic cells (Durand et al., 2014; Wang et al., 2018). Its functions are linked to virulence factors and it delivers bacteriolytic effectors to target cells. In *P. aeruginosa*, it has been reported that the Type VI Secretion System can secrete three types of exported effectors (Tse1-3) that act to destroy cell membranes, peptidoglycans and cytoplasmic components in infected organisms (Russell et al., 2011, 2013). The *VgrG* genes are part of the Type VI Secretion System machinery, also acting as effectors; *VgrG1* possesses a C-terminal extension, defined as an actin cross-linking domain of the *Vibrio cholerae* Type VI Secretion System (Ma et al., 2009).

In the iron uptake category, a number of genes responsible for production of metal binding proteins were identified in both genomic sequences; these include the iron ABC transporter system and the iron-sulfur cluster binding protein. Both are associated with bacterial virulence. The iron ABC transporter permease was also identified as unique gene in the genome sequence of C7. Iron is an important element for survival and colonization by bacteria since it plays a considerable role in the electron transport chain to produce energy (Mietzner and Morse, 1994). Successful competition for iron uptake is therefore crucial for pathogenicity. The privileged iron acquisition system, containing siderophore biosynthesis (*iucABD*/*iutA* operon) and *EfeO* systems for the acquisition of ferrous iron were identified in *C. sakazakii* C7 and C8. This ability may lead to the survival of *C. sakazakii* in blood and thereby its ability to invade the central nervous system (Singh et al., 2015) by crossing the blood-brain barrier. The latter is a characteristic responsible for the worst diseases observed to be caused by *C. sakazakii*.

In the toxin category, many genes associated with toxins such as a *HigB* toxin protein, *RelE*/*StbE* replicon stabilization toxin and *HigA* protein (antitoxin to *HigB*) were identified in the genomic sequence of both C7 and C8. Furthermore, *C. sakazakii* C7 exhibited one unique toxin *RelE* gene associated with cellular processes that include persistence. *RelE* bacterial toxin is structurally like microbial endoribonucleases (Griffin et al., 2013); RelE has been suggested to “represent a shift in the RNase general acid-base catalytic paradigm and promote catalysis predominantly by leaving-group protonation” and charge stabilization (Griffin et al., 2013). In summary, several unique genes associated with pathogenicity and virulence were identified in the genomic sequences of C7 (milk powder) and C8 (ready-to-eat mixed salad) isolates.

#### Phage-Associated Regions

Prophages are mobile genetic elements able to deliver virulence factors (O’Brien et al., 1984) or antimicrobial-resistance genes (Colomer-Lluch et al., 2011) to bacterial hosts and increase the diversity of the host genome (Ventura et al., 2006). We found three regions associated with phages (A1–A3) in the genome of C7. We also identified three regions associated with phages (S1–S3) in the genome of C8 (Supplementary Table S5). Each isolate contains two intact phages. Further, we found one questionable prophage in the S1 region (*Entero*-Tyrion phage) of the C8 genome, whereas for C7 one incomplete prophage was identified.

## Conclusion

In this study, we present the WGS results of *C. sakazakii* strains isolated from ready-to-eat mixed salad and skimmed milk powder. Many identified genes harbored by both isolates are associated with multidrug-resistance, pathogenesis, virulence, and biofilm formation ability. Several identified genes associated with producing capsules and biofilms point to this as an important mechanism for the *C. sakazakii* defense against desiccation and its ability to survive in, e.g., milk powder for a long time. Analyzing all these traits gives a molecular basis to understand their ability to survive the extremely stressful environments met in food production and storage, including the highly desiccated and heat-treated environment of dried milk powder. The genome of *C. sakazakii* isolated in Austria from ready-to-eat mixed salad held a unique ST not previously observed, but also most of the genes implicating *C. sakazakii* as a highly virulent pathogen. The presence of such *C. sakazakii* in ready-to-eat food indicates another potential route for infection and pathogenicity by *C. sakazakii*, besides the established route of powdered milk formulas. Especially disturbing is the multidrug antibiotics resistance found in both isolates. Our study provides new data to better understand the pathogenicity mechanism and virulence of *C. sakazakii* in food ingredients, and to improve monitoring and tracking of the source of food contamination. It highlights the use of WGS for traceability and detection of *C. sakazakii* strains. Based on our findings we call for further research to clarify the potential reservoirs of this emerging pathogen as well as to how and where it has acquired its multidrug antibiotics resistance.

## Data Availability

The datasets generated in this manuscript can be found on NCBI under the *C. sakazakii* complete genome bioproject number PRJNA510032 and biosample accession numbers are SAMN10593105 and SAMN10593106 for *C. sakazakii* C7 and C8, respectively.

## Author Contributions

MA designed and carried out the work. ER and KD supervised the work. ER and MA wrote the manuscript. All authors reviewed the manuscript.

## Conflict of Interest Statement

The authors declare that the research was conducted in the absence of any commercial or financial relationships that could be construed as a potential conflict of interest.

## Footnotes

1 http://pubmlst.org/cronobacter/
